# A Review on Resonant MEMS Electric Field Sensors

**DOI:** 10.3390/mi15111333

**Published:** 2024-10-31

**Authors:** Guijie Wang, Pengfei Yang, Zhaozhi Chu, Lifang Ran, Jianhua Li, Bo Zhang, Xiaolong Wen

**Affiliations:** 1 School of Mathematics and Physics, Beijing Weak Magnetic Testing and Applied Engineering Technology Research Center, University of Science and Technology Beijing, Beijing 100083, China; m202210774@xs.ustb.edu.cn (G.W.); rlf13896842343@163.com (L.R.); jianhuali@ustb.edu.cn (J.L.); bzhang@ustb.edu.cn (B.Z.); 2 School of Applied Science, Beijing Information Science and Technology University, Beijing 100192, China; pfy@bistu.edu.cn; 3 Institute of Microelectronics of Chinese Academy of Sciences, Beijing 100029, China; chuzhaozhi@ime.ac.cn

**Keywords:** electric field sensor, micro-electromechanical systems, MEMS resonant EFS, resonator, mode localization

## Abstract

Electric field sensors (EFSs) are widely used in various fields, particularly in accurately assessing atmospheric electric fields and high-voltage power lines. Precisely detecting electric fields enhances the accuracy of weather forecasting and contributes to the safe operation of power grids. This paper comprehensively reviews the development of micro-electromechanical system (MEMS) resonant EFSs, including theoretical analysis, working principles, and applications. MEMS resonant EFSs have developed into various structures over the past decades. They have been reported to measure electric field strength by detecting changes in the induced charge on the electrodes. Significant advancements include diverse driving and sensing structures, along with improved dynamic range, sensitivity, and resolution. Recently, mode localization has gained attention and has been applied to electric field sensing. This paper reviews the performances and structures of MEMS resonant EFSs over recent decades and highlights recent advances in weakly coupled resonant EFSs, offering comprehensive guidance for researchers.

## 1. Introduction

High-performance micro-electromechanical system (MEMS) electric field sensors (EFSs) have been applied across various fields, including atmospheric electricity, power grids, and biomedical sciences [1,2,3,4,5,6,7,8,9]. In atmospheric electricity, MEMS EFSs monitor atmospheric electric fields, enabling more accurate lightning prediction, analyses of meteorological phenomena, and assessments of electric field impacts on climate change, ultimately improving weather forecasting accuracy. In power grids, MEMS EFSs detect electric field strength around high-voltage power lines, enhancing grid safety and enabling real-time identification of faults or disturbances to ensure system stability and reliability. In biomedicine, they monitor and analyze electric field changes in biological tissues, particularly for electrophysiological research.

The EFSs can be categorized by sensing principles into several types: macroscopic field mill, capacitive EFS, micro-field-mill, frequency-modulated EFS, and mode-localized EFS [10]. The macroscopic field mill excels in atmospheric electric field measurements due to its wide measurement range, high dynamic range, and stability [11,12,13,14]. However, complex structures, maintenance challenges, and large sizes pose obstacles for large-scale sensor networks that require low costs, low power consumption, and high integration. MEMS EFSs address the need for miniaturization while enhancing sensitivity and stability, thereby broadening their applications across various fields.

With the widespread adoption of MEMS technology, MEMS resonators have become a research focus. Researchers have begun applying MEMS resonators to electric field detection, leading to the development of high-performance MEMS resonant EFSs, including micro-field-mills [15], micro-cantilever EFSs [16], frequency-modulated EFSs [17], and mode-localized EFSs [18]. Due to their high-frequency response and small mass, MEMS resonators are widely used in micro-field-mills [19,20,21,22,23,24,25]. Compared to macroscopic field mills, micro-field-mills maintain high performance while offering lower power consumption and more compact size, making them increasingly attractive for electric field measurement. Based on the vibration direction of the resonant elements, micro-field-mills can be classified into three types: in-plane resonant, multi-axis sensitive, and torsional resonant [26,27,28,29,30]. In-plane resonant micro-field-mills work by allowing the resonator to vibrate periodically, exposing the sensing electrodes to the electric field, and generating periodic induced charges to measure the electric field. Multi-axis sensitive micro-field-mills, which provide multi-directional detection and high measurement accuracy, have been successfully validated. The torsional resonant micro-field-mills are recognized for their outstanding charge-sensing efficiency. In comparison, in-plane resonant micro-field-mills offer advantages such as mature processes, lower manufacturing complexity and long-term reliability over multi-axis and torsional resonant micro-field-mills [31,32,33].

Various structures have emerged in the design of MEMS EFSs, each tailored for distinct purposes. These designs are based on Newton’s second law and the principle of electrostatic induction, with the resonator structure forming the core element of these EFSs. The MEMS EFSs have typically evolved into designs featuring electrostatic comb resonators, rotary resonators, micro-cantilever resonators, and torsional resonators. Their respective advantages are low power consumption, multi-directional sensing, fast response time, and high charge induction efficiency [28,29,30,34,35,36,37]. The micro-field-mill, consisting of grounded movable shielding and fixed sensing electrodes, is widely used in electrostatic and atmospheric electric field measurements due to its high sensitivity and stability [38,39,40,41]. And, to meet the demand for high-performance sensors, researchers have conducted extensive studies on packaging and chip integration based on micro-field-mills to enhance sensor performance [42,43,44]. Notably, the integrated micro-field-mill with vacuum packaging has shown great potential in atmospheric electric field monitoring, signaling a significant avenue for future development. In 2015, P.F. Yang et al. developed an atmospheric EFS for lightning hazard warning applications [45]. In recent years, dozens of micro-field-mill designs have been reported. The performance of micro-field-mills has been validated through researchers’ studies on various levels, confirming its claimed advantages. Additionally, micro-field-mills have already been applied in atmospheric electric field tests [46].

Frequency modulation and mode localization in coupled resonators have emerged as new paradigms for electric field sensing [47,48,49,50,51,52,53]. Frequency-modulated EFS measures the electric field by detecting changes in the resonator stiffness caused by the electric field (leading to a frequency shift). Mode-localized EFSs are typically constructed from multi-resonators that are interconnected through weak coupling. The mode localization phenomenon can be summarized as follows: external perturbations affect the resonator stiffness, causing slight asymmetry or detuning in a coupled resonator system. It leads to the redistribution of the resonant energy of the weakly coupled system. This phenomenon is highly sensitive to external perturbations, as the redistribution of energy significantly alters the amplitude of the resonator, thereby amplifying the measurement signal through the amplitude ratio of the resonator [54,55,56,57,58,59]. Charge sensors and electrometers are related to MEMS EFSs in terms of structure, working principles, and the measurements to be conducted; therefore, this review will discuss them together. In 2010, P. Thiruvenkatanathan et al. [18] reported the first mode-localized electrometer.

This paper reviews the different structures, principles, and performances of MEMS EFSs. The MEMS resonant EFSs can be classified based on their resonator structures, sensing directions, underlying principles, output metrics, or distinctive designs. Then, the performance of MEMS resonant EFSs is discussed. This paper is organized as follows: Section 2 describes micro-field-mills; Section 3 reviews frequency-modulated electric field microsensors; Section 4 examines mode-localized electric field microsensors; Section 5 concludes the paper.

## 2. Electric Field Microsensors

### 2.1. Working Principle

**Figure 1:**
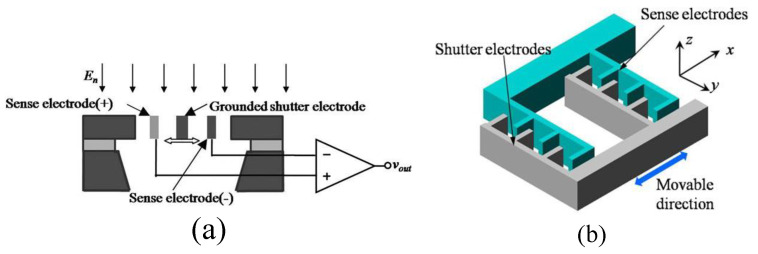
The overall working principle of the micro-field-mill: (**a**) schematic diagram of the principle; (**b**) the corresponding main structural parameters of the reference electrodes and movable structure (modified from [42]).

**Figure 2:**
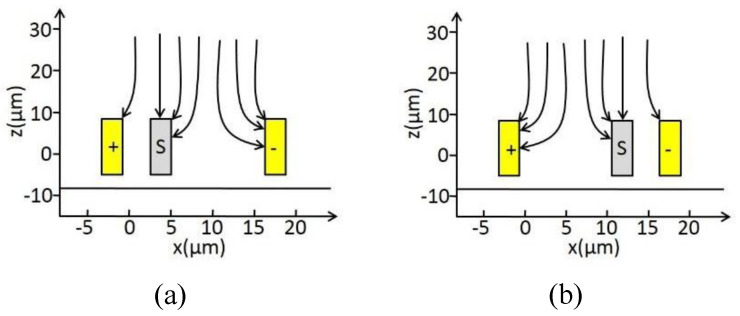
Electric field line distribution after applying the electric field: (**a**) the condition when the shutter is moved near the sensing electrode (+); (**b**) the condition when the shutter is moved near the sensing electrode (−) (after [60], modified).

The main structure of the micro-field-mill includes a grounded movable shielding electrode, a sensing electrode (+), and a sensing electrode (−). The operating principle and the corresponding main structural parameters are shown in Figure 1. The grounded movable shielding electrode undergoes lateral periodic vibration under the external drive. As the shielding electrode moves from one side to the other under the influence of the external electric field $E_{n}$, the distribution of electric field lines changes. As shown in Figure 2, when the shielding electrode is at the far-left position, more electric field lines terminate on the sensing electrode (−) than on the sensing electrode (+), resulting in more induced charges on the sensing electrode (−). The situation is reversed when the shielding electrode moves to the far-right position. According to the principle of charge induction, the induced charge on the sensing electrode can be expressed as:

(1) $$Q\left(t\right)=Q_{A}sin\omega t$$

According to Gauss’s law, the amplitude of charge variation $Q_{A}=f\left(x\right)E_{n}$, where f(x) represents the amplitude of charge variation per kV/m, and experiments have shown that f(x) is proportional to *x* (i.e., $f\left(x\right)=k_{q}x$) [42]. Here, *x* is the resonance amplitude of the shielding electrode, which can be estimated by simulating based on the lumped parameter model. $k_{q}$ represents the conversion coefficient from amplitude per kV/m to charge variation. Under the periodic vibration of the shielding electrode, the induced charge on the sensing electrode can be expressed as an induced current:

(2) $$i_{s}=\frac{dQ(t)}{dt}=k_{q}x\omega E_{n}cos\omega t$$

Assuming the gain of the trans-impedance amplifier (TIA) is $R_{f}$, the induced output voltage *V* and its sensitivity $S_{E}$ of the electric field are:

(3) $$\left\{\begin{array}{c} V=k_{q}\omega xE_{n}R_{f}\\ S_{E}=\frac{dV}{dE}=k_{q}\omega xR_{f} \end{array}\right.$$

### 2.2. Lumped Model Analysis

**Figure 3:**
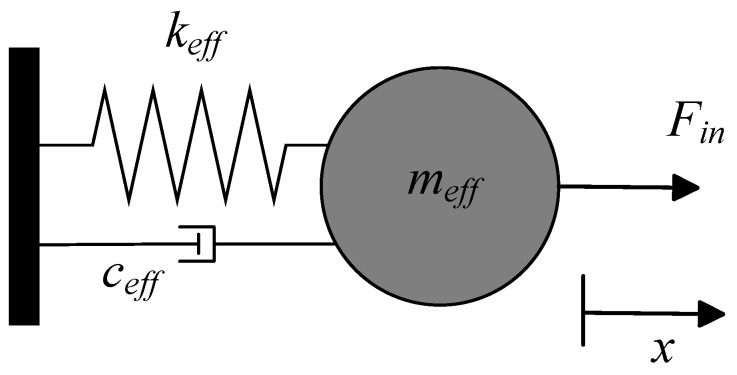
Lumped mass-spring-dashpot model corresponding to 1-DOF resonant system.

The working principle of the micro-field-mills can be equivalently described using a lumped parameter model, either as a mechanical mass-spring-dashpot system or as an electrical Butterworth–Van Dyke (BVD) model. Firstly, the mass-spring-dashpot model represents the simplest resonator model, as shown in Figure 3. Based on Newton’s laws of motion, the relationship between the displacement of the mass block and the input force can be derived using the following equation:

(4) $$m\ddot{x}+c\dot{x}+kx=f\left(t\right)$$

where *m* represents the effective mass of the resonator, *k* is the effective stiffness, *c* is the effective damping, and f(t) is the driving force. In the Laplace domain, this equation is expressed as:

(5) $$ms^{2}X\left(s\right)+csX\left(s\right)+kX\left(s\right)=F\left(s\right)$$

where *s* is the complex domain frequency. The transfer function can be expressed as:

(6) $$H_{1}\left(s\right)=\frac{X(s)}{F(s)}=\frac{1}{ms^{2}+cs+k}=\frac{1}{k}\left(\frac{\omega_{0}^{2}}{s^{2}+\frac{\omega_{0}}{Q}s+\omega_{0}^{2}}\right)$$

where $\omega_{0}$ is the ideal undamped resonant frequency of the system (Eigen-frequency) and Q is the Q-factor. For an ideal undamped second-order system, the Eigen-frequency is:

(7) $$\omega_{0}=2\pi f_{0}=\sqrt{\frac{k}{m}}$$

Under an external electric field, the transfer function of the output from the resonant micro-field-mill’s sensitive structure can be expressed as:

(8) $$H\left(s\right)=\frac{I(s)}{F(s)}=\frac{I(s)}{X(s)}\cdot \frac{X(s)}{F(s)}=H_{2}\left(s\right)\cdot H_{1}\left(s\right)$$

where $H_{2}\left(s\right)=I\left(s\right)/X\left(s\right)$, and based on Equation (2), it can be derived that:

(9) $$H_{2}\left(s\right)=\frac{I(s)}{X(s)}=\frac{(k_{q}\omega_{0}E_{n})s}{s^{2}+\omega_{0}^{2}}$$

where

(10) $$H\left(s\right)=\frac{I(s)}{F(s)}=\frac{(k_{q}\omega_{0}^{3}E_{n})s}{k\left(s^{2}+\omega_{0}^{2}\right)\left(s^{2}+\frac{\omega_{0}}{Q}s+\omega_{0}^{2}\right)}$$

**Figure 4:**
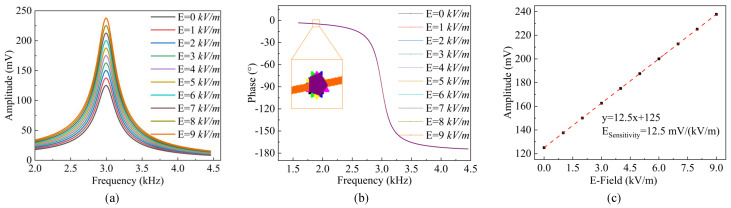
Amplitude–frequency response (**a**), phase–frequency response (**b**), and sensitivity curves (**c**) from numerical simulations based on the structural parameters of the micro-field-mill device, as described in Reference [46].

Based on the micro-field-mill device structural parameters described in Reference [46], numerical simulations were performed using the transfer function equation from Equation (10). The simulation results show the frequency response under different electric field strengths, as illustrated in Figure 4a,b. The amplitude of the resonator increases with the rising electric field strength. By varying the electric field strength, the simulated electric field sensitivity of the structure was determined to be 12.5 mV/(kV/m), as shown in Figure 4c. These results demonstrate that the structure exhibits good response characteristics and sensitivity under varying electric field conditions.

**Figure 5:**
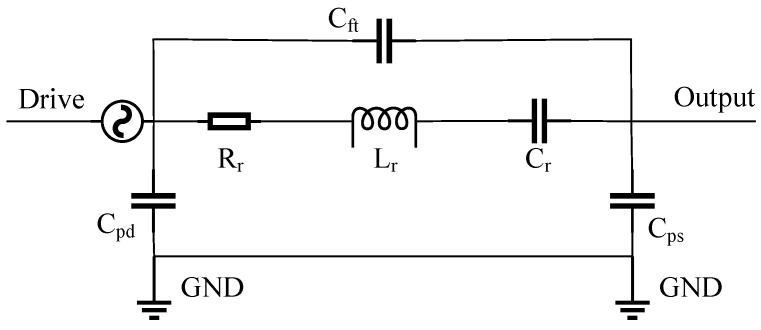
BVD model corresponding to a 1-DOF resonant system.

In the application of MEMS sensors, MEMS resonators typically vibrate at the driving force. The amplitude of the resonator is then detected as an output signal and converted into an electrical signal through various techniques such as front-end amplification circuits or instruments. By analogizing the electrical and mechanical resonator models, an equivalent RLC circuit model of the MEMS resonator is constructed. This model facilitates synchronous simulation of MEMS resonators in electrical and mechanical domains, typically using LTspice 24.0.9 simulation software. Based on the structural parameters of the MEMS resonator from Reference [46], an equivalent BVD model of the 1-degree-of-freedom (DOF) resonant system, including various parasitic capacitances that cause leakage current, is established, as shown in Figure 5. In this model, $R_{r}$, $L_{r}$, and $C_{r}$ represent resistance, inductance, and capacitance in the circuit, respectively. $C_{pd}$, $C_{ps}$, and $C_{ft}$ represent the parasitic capacitances from the drive and sense pads to the grounded substrate and the feedthrough capacitance from the drive port to the sense port via the resonator body. Table 1 lists the corresponding relationships between the parameters of the mechanical and electrical systems.

In the equivalent BVD model of the 1-DOF resonant system shown in Figure 5, the effective impedance ($Z_{R}$) and transfer function of the resonator part ($Y_{R}$) can be expressed as:

(11) $$\left\{\begin{array}{c} Z_{R}=R_{r}+\frac{1}{j\omega C_{r}}+j\omega L_{r}\\ Y_{R}=\frac{i(j\omega )}{v(j\omega )}=\frac{1}{R_{r}+\frac{1}{j\omega C_{r}}+j\omega L_{r}} \end{array}\right.$$

Based on the transfer function of the 1-DOF resonator, the equivalent spring, mass, and damping values can be transformed from the mechanical domain to the electrical domain as follows:

(12a) $$\begin{array}{c} R_{r}=\frac{\sqrt{km}}{Q\eta^{2}} \end{array}$$

(12b) $$\begin{array}{c} L_{r}=\frac{m}{\eta^{2}} \end{array}$$

(12c) $$\begin{array}{c} C_{r}=\frac{\eta^{2}}{k} \end{array}$$

where *m* and *k* denote the effective mass and the effective stiffness of the resonators, respectively; *Q* represents the Q-factor associated with the damping constant; and η is the electro-mechanical transduction coefficient. For the structure described in Reference [46], the system is a symmetric capacitive transducer, where the electro-mechanical coupling coefficient is determined by the electrode area *A*, electrode gap *g*, drive voltage *V* and the permittivity ϵ of the working medium. It can be described as:

(13) $$\eta =\frac{\epsilon AV}{g^{2}}$$

**Figure 6:**
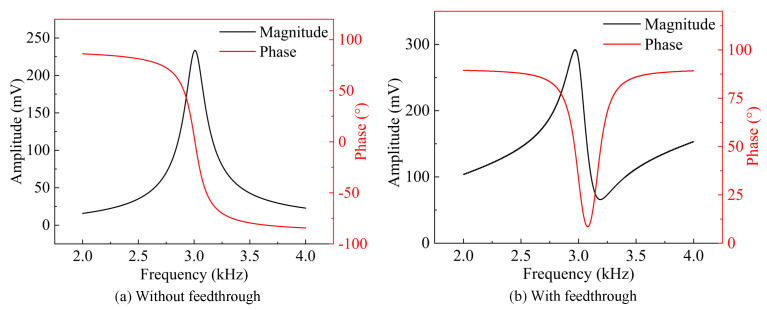
Frequency response simulation based on the 1-DOF BVD model.

In the BVD model, the effect of parasitic capacitance is usually negligible compared to the significant impact of feedthrough capacitance [54]. Therefore, only the effect of the feedthrough capacitance $C_{ft}$ is considered when simulating the BVD model based on the structural parameters. The circuit shown in Figure 5 is simulated using LTspice software, and the magnitude–frequency response is compared with and without the presence of feedthrough capacitance, as illustrated in Figure 6. From the simulation results, it is evident that the feedthrough current induces an anti-resonance peak in the frequency response of the resonator. The occurrence of this anti-resonance appears before or after the resonance peak—depending on the direction of the feedthrough current. The total output current of the resonator can be expressed as:

(14) $$\begin{array}{c} i_{0}=i_{r}+i_{f}=\frac{v(j\omega )}{Z_{R}//Z_{ft}}\\ =v\left(j\omega \right)\frac{\left[1-\omega^{2}C_{ft}L_{r}+\frac{C_{ft}}{C_{r}}+j\omega R_{r}C_{ft}\right]}{R_{r}+j\left(\omega L_{r}-\frac{1}{\omega C_{r}}\right)}\\ =\frac{\left(V_{bias}+V_{ac}\right)\left[R_{r}+j\left(\omega R_{r}^{2}C_{ft}-\epsilon \Psi \right)\right]}{R_{r}^{2}+\psi^{2}} \end{array}$$

where $i_{r}$ represents the effective motion current, which is the current signal caused by the movement of the sensing capacitor, while $i_{ft}$ represents the driving feedthrough current. The feedthrough capacitance determines the magnitude of the feedthrough current. And $\epsilon =1-\omega^{2}C_{ft}L_{r}+C_{ft}/C_{r}$, $\psi =(\omega L_{r}-1/\omega C_{r}$). At the resonator’s Eigen-frequency ($\omega =\sqrt{k/m}=\sqrt{1/L_{r}C_{r}}$), the output current of the resonator can be expressed as:

(15a) $$\begin{array}{c} |i_{0}|\propto \sqrt{1+C_{ft}^{2}/Q^{2}C_{r}^{2}}/R_{r} \end{array}$$

(15b) $$\begin{array}{c} \varphi =arctan(\frac{C_{ft}}{QC_{r}}) \end{array}$$

It can be observed that: (1) the feedthrough current affects both the amplitude and phase of the resonator’s output current; (2) the larger the Q-factor, the smaller the impact of the feedthrough current. Therefore, the impact of feedthrough capacitance must be considered when designing MEMS resonance devices.

### 2.3. Micro-Field-Mills

**Figure 7:**
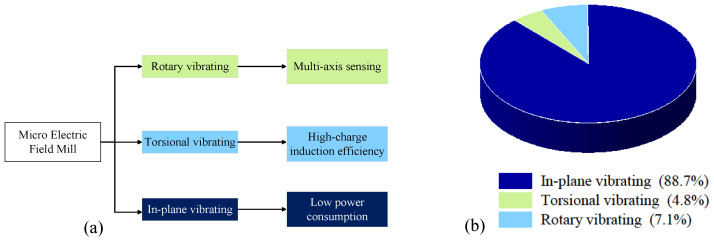
Classification (**a**) and proportion (**b**) of micro-field-mills based on different vibration modes and structures. Among the micro-field-mills reviewed, more than 80% of micro-field-mills utilize the classic in-plane vibrating structure, which is the most widely used design.

As shown in Figure 7, the surveyed micro-field-mills show that 88.7% are in-plane vibrating types, 4.8% are torsional, and 7.1% are rotary. The classic in-plane vibrating micro-field-mills dominate the market due to their simple structure, low power consumption and costs. In addition, the micro-field-mills developed by X.L. Wen et al. for measuring atmospheric electric fields and static electricity have been commercialized [46].

Several typical in-plane vibrating micro-field-mill structures have been developed to achieve high-precision electric field measurements over the past three decades. These structures are typically fabricated using Silicon-on-Insulator (SOI) technology. The main components consist of various types of resonant beams, proof masses, and sensing electrodes that interact with the electric field. The sensors typically use capacitive sensing mechanisms, such as parallel plate or comb-finger structures, to detect changes in capacitance. Other key components include comb-drive electrodes for resonator actuation, shielding electrodes with a movable shutter, and vacuum packaging for stability enhancement. Several optimizations have been implemented in the design of these micro-field-mills to enhance electric field measurement performance. The key structural variations include folded beams, double-clamped thin beams, parallel plate capacitive membrane sensing structures, and comb-finger capacitive sliding film sensing structures.

#### 2.3.1. Folded Beam Micro-Field-Mills

**Figure 8:**
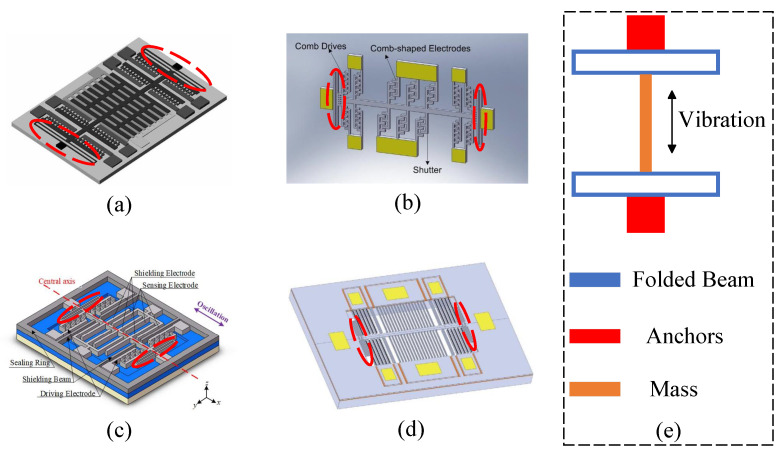
Four micro-field-mill structures using folded beams, with (**a**–**d**) corresponding to [24,62,63,64], respectively, and a schematic of the folded beam (**e**).

A resonator with folded beams typically features a central mass, as depicted in Figure 8e. The two ends of the mass are connected to rectangular folded beams (spring beams), which are anchored at their opposite ends. Spring beams have been a fundamental part of micro-field-mill design from the beginning, and over 60% of the reviewed micro-field-mills utilize this folded beam configuration. In 1991, C.H. Hsu et al. first reported an electrostatic voltmeter utilizing a folded beam as a key element of the voltmeter, demonstrating a sensitivity of 20 μV/V [61]. P.S. Riehl et al. reported a similar structure for an electrostatic charge and micro-field-mill in 2003 [19], which improved the SNR. This design employed a combination of fluid self-assembly JFET and SOI microstructure technology and integration techniques involving CMOS and polysilicon. It achieved a charge resolution of 4.5 aC rms within a 0.3 Hz bandwidth and an electric field resolution of 630 V/m, showcasing some of the best performance data available at the time.

In 2006, C.R. Peng et al. proposed a micro-field-mill structure for atmospheric electric field detection [24], fabricated using a three-layer polysilicon surface micromachining process, as shown in Figure 8a. Fixed with a 25 V direct current (DC) and a 0.3 V AC drive signal, the sensor exhibited its highest sensitivity at a working frequency of 4.13 kHz. Testing revealed an electric field resolution of less than 100 V/m and an uncertainty of less than 5%. In the same year, they conducted a more detailed characterization of the structure [25], achieving the best resolution performance for a MEMS-based EFS in ambient air at room temperature, with a resolution of 200 V/m, and attained a nonlinearity of 1.8% within the measurement range of 0–10 kV/m. Subsequently, X.X. Chen et al. reported a thermally actuated micro-field-mill structure [26] that utilized a compact bent-beam thermal actuator. The driving method can achieve a drive voltage amplitude of ±2 V, and the micro-field-mill achieves a resolution of 240.8 V/m at a working frequency of 40 kHz.

**Figure 9:**
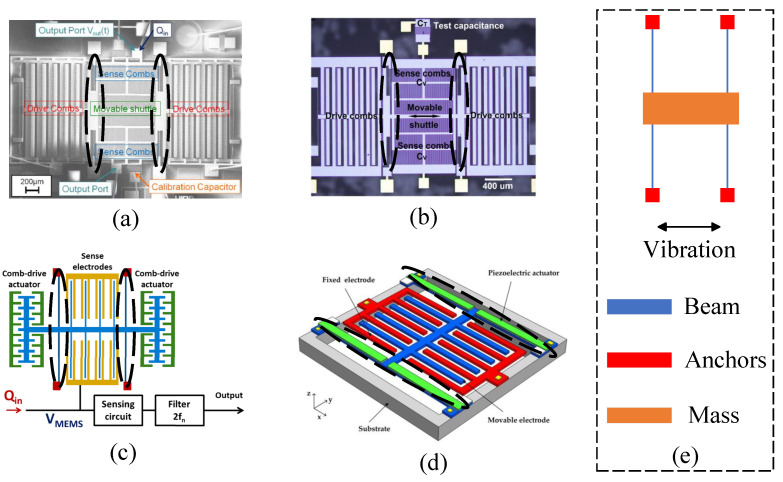
Four micro-field-mill structures using Double-clamped beams, with (**a**–**d**) corresponding to [39,43,65,66], respectively, and a schematic of the Double-clamped beam (**e**).

B. Bahreyni et al. reported a micro-field-mill structure in 2008 [32], as shown in Figure 9b, which employs multiple folded beams and a thermal actuator to drive the shutter. This design effectively reduces the amplitude of the driving signal and minimizes signal interference. The sensor achieved a resolution of 42 V/m, representing a significant improvement over previous sensors. In 2010, C.R. Reng et al. proposed a micro-field-mill suitable for electrical engineering applications [35]. Under a vacuum of approximately 1 mTorr and with a low driving voltage, this sensor achieved a Q-factor of 31,034. The electric field measurement range reached 50 kV/m, with a resolution better than 50 V/m. In 2015, P.F. Yang et al. reported a micro-field-mill designed for lightning hazard warning, which is an optimized version of previously reported structures. This sensor boasts excellent performance metrics, including a minimum detectable electric field of 10 V/m, an uncertainty of 0.67% over a range of 0 to 50 kV/m, and power consumption of only 0.62 W.

In 2021, X.L. Wen et al. reported a type of MEMS-based electric field sensor utilizing a spring beam as the resonant element, as shown in Figure 8b [62]. This micro-field-mill has a measurement range of −30–30 kV/m, with a power consumption of 0.6 W at 1 Hz, and operates within a temperature range of −40–55 ^∘^C. The electric field resolution is approximately 5 V/m, and the RH range (%RH) is 0–100. C.R. Peng et al. (2022) reported a micro-field-mill structure [63] as shown in Figure 8c. This is a novel wafer-level vacuum-packaged micro-field-mill that offers a higher Q-factor and lower power consumption compared to previously reported micro-field-mills. The micro-field-mill utilizes a differential amplifier circuit to enhance the SNR, with a natural frequency of approximately 5369 Hz, and the micro-field-mill features a measurement range of 0–50 kV/m with a sensitivity of 0.16 mV/(kV/m). It maintains a Q-factor greater than 5000, showing no decline over a 50-day testing period. Furthermore, the microsensor demonstrates a linearity of 1.62% and an uncertainty of 4.42%. In 2023, S.P. Chen et al. reported a micro-field-mill composed of folded beams that operate under atmospheric pressure, as shown in Figure 8d [64]. Using a coupled electrostatic-flow-structural 3D finite element model, the resonant displacement is directly calculated, accounting for changes in the system’s effective mass and resonant frequency due to variations in structural parameters. The experimental results demonstrate a significant performance improvement.

#### 2.3.2. Double-Clamped Beam Micro-Field-Mills

Micro-field-mills composed of double-clamped beams generally include two such beams, with both ends of the beams anchored and a central mass (fitted with the electrode device) connected between them, as shown in Figure 9e. The double-clamped beam design emerged earlier in micro-field-mills, and while similar research continues, it is less frequently reported compared to folded beam designs. In 2004, C. Gong et al. first proposed two types of micro-field-mill structures based on double-clamped beams, one utilizing a parallel vibration mode and the other a vertical vibration mode. Simulations and comparisons of the two structures showed that the induced currents in both designs are measurable [20]. Y. Zhu et al. reported a resonant MEMS electrostatic charge sensor [33] in 2008 similar to the one shown in Figure 9c, which modulates the DC charge to be measured and converts it into an AC voltage output, thereby improving the SNR. In an air environment, the sensor operates at a frequency of 3.2 kHz, achieving a charge-to-voltage conversion gain of 2.06 nV/e and demonstrating an excellent background noise performance of 52.4 e/rtHz. A. Menzel et al. studied a sensor for detecting biomolecules and electrochemical charges in 2011 [39], as shown in Figure 9a. Under room temperature and ambient pressure conditions, this micromechanical electrometer demonstrated a resolution of 3 e/$\sqrt{\mathrm{Hz}}$ and the ability to detect single-charged molecules bound to the electrode surface.

In 2013, G. Jaramillo et al. reported an electrometer utilizing two double beams as the resonant elements [43], with its structural schematic diagram shown in Figure 9c. This electrometer achieved approximately three times improvement in resonator responsivity through an optimized circuit layout, with a resonant frequency of 2.3 kHz. By reducing the electrode size, the electrometer lowered the capacitance of the porous sampling electrode to 2–3 pF, achieving a resolution of approximately 1 fA and a 5-fold increase in sensitivity.

J. Jalil et al. reported a high-sensitivity and high-resolution electrostatic testing system based on a double-clamped beam resonator in 2018 [65], utilizing Silicon-on-Glass (SOG) MEMS technology, as shown in Figure 9b. Replacing the silicon substrate with a glass substrate effectively reduced parasitic capacitance, significantly enhancing charge sensitivity. The system achieved a sensitivity of $1.43\times 10^{11}$ V/C with an improved amplification circuit. Additionally, it demonstrated an optimal charge resolution of 1.03 e/$\sqrt{\mathrm{Hz}}$ at 5.7 kHz under room temperature and atmospheric pressure conditions. In 2021, H.C. Lei et al. reported a type of micro-field-mill with a resonant element consisting of two double-clamped thin beams and a mass, utilizing a piezoelectric drive, as indicated in Figure 9d [66]. In this micro-field-mill, the mutually shielded electrodes function both as sensing and shielding electrodes, with the movable electrode being driven vertically by piezoelectric means. This mutual shielding design effectively doubles the sensing area of the micro-field-mill, ultimately enhancing the sensor’s sensitivity.

#### 2.3.3. Parallel Plate Capacitive Membrane Sensing Micro-Field-Mills

**Figure 10:**
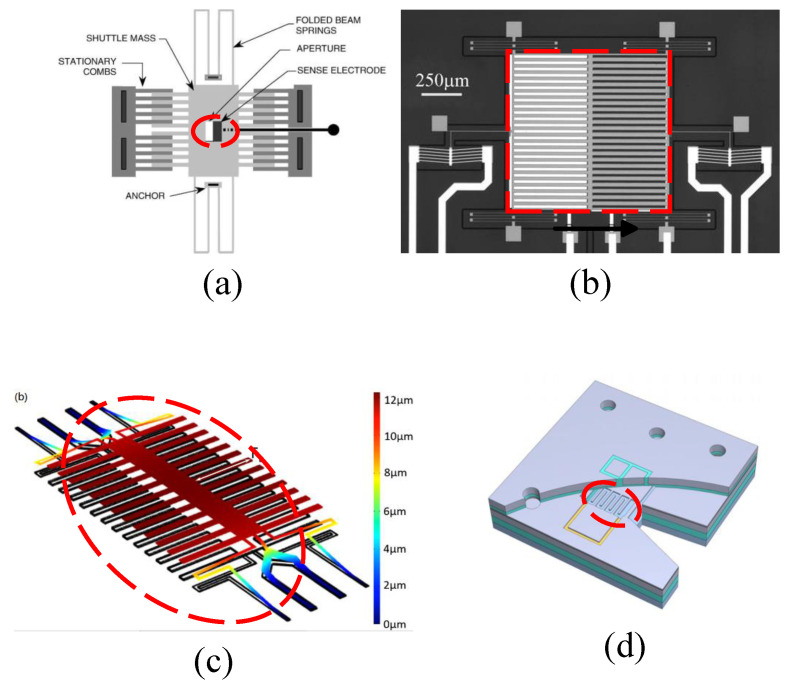
Four micro-field-millstructures using Parallel-plate capacitive membrane sensing structures, with (**a**–**d**) corresponding to [15,32,44], respectively.

Early micro-field-mills mostly used parallel plate capacitors for membrane sensing due to their structural simplicity and reliable long-term stability. As early as 2001, M. N. Horenstein et al. reported a micro-aperture electrostatic field mill using parallel plate capacitive membrane sensing [15], as shown in Figure 10a. The device was constructed using a silicon surface micromachined MEMS fabrication process. The micro-aperture refers to a moving shuttle with a 10 μm × 10 μm observation aperture that periodically exposes and covers the charge-sensing electrodes, enabling the measurement of electrostatic field strength. This micro-field-mill is driven by an AC actuator, operating stably at its natural frequency of 7.6 kHz. The sensor exhibits a sensitivity of 35 μV/(kV/m) and offers an ultra-wide measurement range of 500 kV/m.

In 2006, C.R. Peng et al. reported a design of a resonant miniature electrostatic field sensor with feedback driving and detection [23], which has a structure similar to that shown in Figure 8a. This design uses closed-loop control, enabling the sensor to self-oscillate at its resonant frequency of 4.13 kHz. The main demodulation function is achieved using a digital lock-in amplifier (LIA), and a nonlinearity of 1.8% was measured within a measurement range of 0–10 kV/m. In the same year, T. Denison et al. introduced a self-resonant MEMS-based electrostatic field sensor [22] with a sensitivity of 4 V/m/$\sqrt{\mathrm{Hz}}$, featuring a similar structure. The design primarily consists of three modules: a MEMS shutter, a sensing interface, and a self-resonant circuit that feeds back to the MEMS shutter, all designed to maximize electric field sensitivity. Test results showed that the sensor achieved high sensitivity, with a reference input noise of 4 V/m/$\sqrt{\mathrm{Hz}}$, a measurement range of −700 kV/m to 700 kV/m, and an RMS field error of less than 40 V/m.

A schematic of a field-chopping electric field sensor based on thermal actuators with mechanically amplified response reported by B. Bahreyni et al. [27] in 2007 is shown in Figure 10b. In this design, an incident electric field is chopped using a perforated shutter, and the induced charges on two sets of electrodes beneath the shutter are differentially measured to detect the electric field. Thermal actuators are used to maintain the structure in resonance, reducing the drive requirements. A resolution of 42 V/m was achieved under good linearity conditions. The following year, X.X. Chen et al. introduced a thermally driven resonant miniature electric field sensor with feedback control [31]. The sensor, excited by a 20 kHz square wave and with a natural frequency of 40 kHz, achieved a resolution of 101.7 V/m and a sensitivity of 98.32 μV/(kV/m).

T. Chen et al. reported a new micro-field-mill structure for measuring a DC electric field in 2014 [44]. This micro-field-mill uses thermal actuators to vertically drive a shutter, periodically modulating the charge on the underlying sensing electrodes to measure the amplitude of the DC electric field. This new structure compensates for the interference from high voltage actuators and the displacement of the shutter under strong electric fields by adjusting the vertical movement of the shutter. Simulation results show that an output current of approximately 1 pA can be achieved with the shutter operating at 7 kHz under a 1 kV/m DC electric field. In 2018, A. Kainz et al. developed a passive optomechanical electric field strength sensor with built-in vibration suppression. This sensor features a suspension system designed to effectively suppress vibration cross-sensitivity and achieves distortion-free and temperature-stable measurements. Results show that the sensor has a resolution of 737 V/m/$\sqrt{\mathrm{Hz}}$, with a theoretical limit resolution of 59.3 V/m/$\sqrt{\mathrm{Hz}}$.

Vacuum packaging technology effectively reduces air damping, minimizes energy loss, and increases the Q-factor of the device, enabling the resonator to operate with greater amplitude and for longer durations. This not only enhances the dynamic response of the sensor but also significantly improves the overall performance of resonant sensors and extends their lifespan. Consequently, vacuum packaging technology has been widely adopted in the design of resonant MEMS EFSs. Most existing studies have focused on horizontal resonant structures, whereas vertical resonant MEMS EFS can significantly enhance sensor sensitivity by modulating the electric field distribution on the sidewalls and top surfaces of sensing electrodes. Developing wafer-level vacuum packaging technology for vertical resonant micro-field-mills is expected to further optimize sensor performance and improve the accuracy and stability of electric field detection.

As shown in Figure 10d, Y.H. Gao et al. reported a wafer-level vacuum-packaged vertical resonant electric field microsensor that uses a three-layer stack structure of glass in silicon anode bonding, SOI, and glass on silicon (GIS-SOI-GOS) in 2024 [67]. In this new structure, GIS is used to fabricate the external drive electrodes for driving the resonator, SOI is used to create the sensitive part of the device layer, and GOS is used for the electric field signal conversion part. Under a pressure of 5 pA, with a fixed 7 V DC voltage and a 70 mV AC voltage, the resonator operates stably at its natural frequency of 5648.9 Hz. Within a measurement range of 0–50 kV/m, the sensor achieves a sensitivity of 0.31 mV/(kV/m), a linearity of 5.84%, a Q-factor of 5071, and a resolution of 230 V/m. To enhance the sensitivity of EFSs, Y.H. Gao et al. subsequently proposed an innovative vertical resonant MEMS electric field sensor based on Through Glass Via (TGV) technology [68]. This microsensor consists of an electric field sensing cap, a driving cap, and an SOI-based microstructure between them. Within a measurement range of 0–50 kV/m, the sensor achieved a sensitivity of 0.82 mV/(kV/m) and a linearity of 0.65%, which is more than twice the sensitivity compared to their previous work [63].

#### 2.3.4. Comb-Finger Capacitive Sliding Film Sensing Micro-Field-Mills

To overcome the limitations of parallel plate capacitive membrane sensing structures, such as high air damping and limited displacement range, the comb-finger capacitive sliding film sensing structure is another commonly used design. This structure alters the capacitance through a pair of interdigitated comb electrodes that slide relative to each other, offering a larger linear displacement range and greater stability. In 2005, C. Gong et al. reported a novel miniature interlacing vibrating EFS [21]. This sensor utilizes a comb-finger structure for both the sensing and shielding electrodes, significantly enhancing the sensing signal. It achieves large displacements under a low driving voltage of 2 V, effectively reducing crosstalk interference. The design offers good dynamic stability, a high SNR, and a high resolution of 100 V/m.

**Figure 11:**
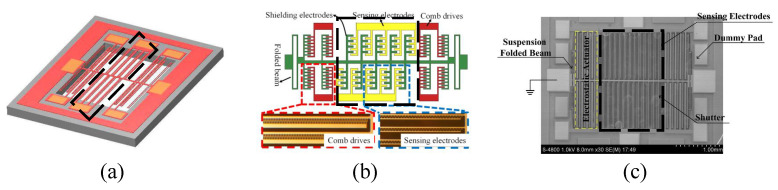
Three micro-field-mill structures using Comb-finger capacitive sliding film sensing structures, with (**a**–**c**) corresponding to [34,46], respectively.

The schematic of the novel closed-loop SOI MEMS resonant electrostatic field sensor [34] reported by C.R. Peng et al. is shown in Figure 11a. The sensing electrodes use a differential comb-finger structure consisting of a positive sensing electrode (+) and a negative sensing electrode (−) to achieve maximum sensitivity to the electric field. Within a measurement range of 0–50 kV/m, the sensor achieved a resolution of 50 V/m, with uncertainty increased by 2.1%. In 2011, P.F. Yang et al. developed a high-sensitivity EFS with a novel comb-shaped microelectrode based on this structure [38]. The introduction of the comb-shaped microelectrode improved the charge-sensing efficiency. Test results in an air environment showed a resolution of 40 V/m and an uncertainty of 1%. Compared to previous designs, the performance was enhanced, and the sensor was successfully used to detect ice formation on high-power cables. Later, they reported an SOI-based resonant electric field microsensor with coplanar comb-shaped electrodes [42]. Using this structure, they proposed a demodulation method for AC and DC electric fields [60] and a non-invasive DC voltage measurement method [69], achieving an electric field measurement range of 0–667 kV/m and a voltage measurement range of −1000 V to 1000 V, respectively. In 2021, X.L. Wen et al. reported a resolution-enhancing structure for an electric field microsensor chip [46], as shown in Figure 11b, and applied it to atmospheric electric field research [70]. This structure achieved a sensitivity of 465 mV/kV/m and a resolution better than 10 V/m.

### 2.4. Torsional and Rotary Vibrating MEMS EFSs

**Figure 12:**
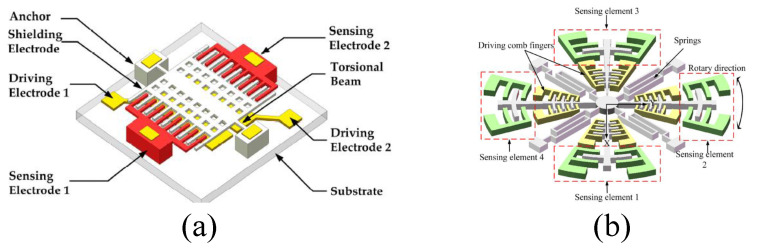
Schematic diagrams of torsional vibration (**a**) (after [30], modified) and rotary vibration (**b**) (after [28,30], modified) in micro electric field mills.

A novel micro-field-mill structure based on torsional resonance [30] was proposed by Z.Z. Chu et al. in 2017, and its schematic is shown in Figure 12a. The structure mainly consists of a torsional shutter composed of shielding electrodes and torsional beams, along with two fixed sensing electrodes. The torsional shutter is driven by a push–pull electrostatic mechanism. Its working principle is similar to that of traditional micro-field-mill designs, where two identical sensing electrode arrays are symmetrically distributed on both sides of the grounded shielding electrode. The torsional shielding electrode and the sensing electrodes are arranged in the same plane in an interdigitated configuration. When the sensor is placed in a vertical electric field, the two sensing electrodes generate induced charges. According to Gauss’s law, the amount of induced charge can also be determined. As the torsional shutter vibrates periodically, the charges on the sensing electrodes vary periodically, generating an induced current. Simulation results indicate that the torsional shutter exhibits a high charge-sensing efficiency, with an experimentally measured efficiency of 48.19 pA/(kV/m). Within a measurement range of 0–50 kV/m, the structure achieves a sensitivity of 4.82 mV/(kV/m) and a linearity of 0.15%. Compared to previously reported micro-field-mills, these performance metrics show a significant improvement.

Y. Wang et al. reported a novel X-Y biaxial EFS based on in-plane rotational vibration [28,29] in 2014, with its structural schematic shown in Figure 12b. The sensor consists of serpentine springs, angular drive comb fingers, and differential sensing elements, allowing for large in-plane rotations, which enhance vibration amplitude and generate more induced charges. Two pairs of sensing elements are positioned differently, forming a cross shape, with each pair sensing the X and Y components of the electric field, respectively. The resonator operates at a frequency of 799 Hz, and within a measurement range of 0–25 kV/m, it achieves an X-axis sensitivity of 0.675 mV/(kV/m), a Y-axis sensitivity of 0.689 mV/(kV/m), and demonstrates good linearity.

### 2.5. Micro-Cantilever EFSs

**Figure 13:**
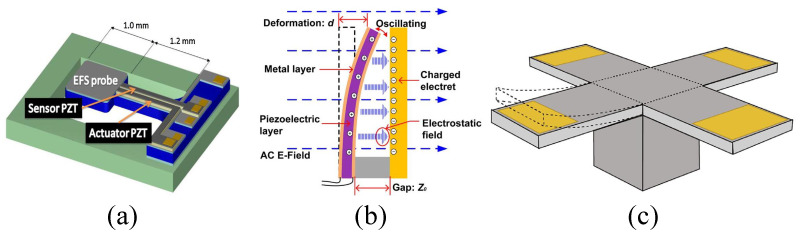
Three micro-field-mill structures based on different Optimized cantilever beams driven by PZT, with (**a**–**c**) corresponding to [36,37,71,72], respectively.

As shown in Figure 13, various types of cantilever beam structures based on piezoelectric and electrostatic actuation have been proposed over the past 20 years due to their simple structure, light weight, and high rigidity, which contribute to low energy consumption. Between 2008 and 2013, T. Kobayashi et al. proposed and developed a piezoelectric-driven MEMS cantilever beam EFS [16,36,37]. This sensor consists of a probe for detecting electrostatic fields and a self-sensitive piezoelectric micro-cantilever beam with a PbO3 (PZT) thin film used for sensing and actuation. At a resonance frequency of 1875 Hz, it exhibits good linearity within a measurement range of −3 kV to 3 kV. In 2015, J.A. Huang et al. reported a high-sensitivity EFS for low-frequency AC field measurements based on a piezoelectric cantilever beam [71] with an electrostatic bias, as shown in Figure 13b. The sensor comprises a polyvinylidene fluoride (PVDF) piezoelectric cantilever beam and a polytetrafluoroethylene (PTFE) electrode. Theoretical analysis indicates that this structure has higher sensitivity. Experimental results show that the sensor operates at a natural frequency of 25.8 Hz, with a measurement range of 180 kV/m and a sensitivity of 0.84 mV/(kV/m).

In 2023, Z.F. Han et al. reported a micro-cantilever beam EFS based on electrostatic actuation and piezoresistive sensing [72]. As shown in Figure 13c, this sensor utilizes four cantilever beam structures that undergo displacement under the influence of electrostatic forces. The resulting strain is converted into a measurable signal through piezoresistive materials, enabling electric field measurement. Experimental results indicate that this EFS features a wide measurement range from 1.1 kV/m to 1100 kV/m, a resolution of 112 V/m$\cdot \sqrt{\mathrm{Hz}}$, a cutoff frequency of 496 Hz, and a high SNR, demonstrating excellent performance.

## 3. Frequency-Modulated Electric Field Microsensors

This section focuses on high-sensitivity electric field measurements based on frequency modulation. Frequency shift output sensors offer several advantages, including enhanced stability, compact size, and low power consumption. First, the vibration theory and sensing principles of frequency shift sensors are introduced. Then, measurement schemes and sensor prototypes for axial and lateral stiffness perturbations are reviewed, including a lateral perturbation electrostatic meter based on a single-anchored circular beam resonator and a parallel plate capacitor transducer. Developing high-performance micro-electrometers facilitates further research on more precise voltmeters and ammeters.

### 3.1. Vibration System and Working Principle

The resonator is the key component of sensors that use frequency shift as the output metric. Mechanical resonators are favored for their high Q-factor, which allows the input energy to be confined within a narrow bandwidth, resulting in excellent SNR and frequency stability. For frequency-modulated sensors, the primary working principle is that an external input to be measured causes a change in the stiffness of the resonator, leading to a shift in its resonant frequency. This frequency shift varies linearly with the measured input, enabling high-sensitivity sensing. This section introduces the resonance principle of resonators and the primary working mechanism of frequency shift sensors.

#### 3.1.1. Resonance Overview

**Figure 14:**
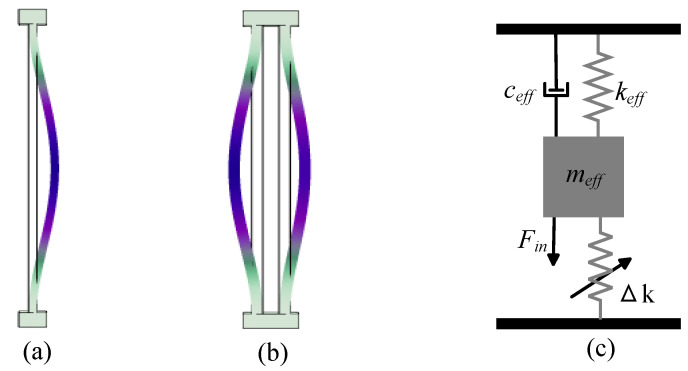
In-plane flexural vibration modes of a single beam resonator (**a**) and a tuning fork resonator (**b**), along with a mass-spring-dashpot model used to describe the resonance of mechanical resonators (**c**).

In a mechanical resonance system, the resonator is an energy storage unit that continuously converts energy between kinetic and potential forms. Each resonant frequency of the resonator corresponds to a different vibration mode. In real-world physical systems, energy dissipation is always present. For resonant motion, whether a simple single-beam resonator or a complex tuning fork resonator, a bending mechanical beam can be described as a mass-spring-dashpot vibration system, as shown in Figure 14. Using Newton’s laws of motion, the dynamic response of a 1-DOF mechanical resonator can be expressed as:

(16) $$m_{eff}\ddot{x}\left(t\right)+c_{eff}\dot{x}\left(t\right)+\left(k_{eff}+\Delta k\right)x\left(t\right)=F\left(t\right)$$

where $m_{eff}$, $c_{eff}$, and $k_{eff}$ represent the effective mass, damping coefficient, and stiffness of the resonator, respectively. x(t) and F(t) denote the displacement of the resonator and the driving force as functions of time. Assume that the vibration of the resonator is harmonic, $x\left(t\right)=x_{A}sin(\omega_{0}t-\phi$), where *t* is time and $x_{A}$ is the amplitude at the Eigen-frequency; ϕ is the phase determined by the initial state of the resonator. Assuming the driving force F(t) balances the system’s damping to maintain resonance, and in the absence of any perturbation, the equation reduces to:

(17) $$m_{eff}\ddot{x}\left(t\right)+k_{eff}x\left(t\right)=0$$

Then, by substituting the harmonic displacement x(t) into Equation (17), we obtain the analytical solution for the natural angular frequency $\omega_{0}^{unperturbed}$:

(18) $$\omega_{0}^{unperturbed}=\sqrt{\frac{k_{eff}}{m_{eff}}}$$

The equation indicates that a resonator’s frequency response can be modulated by altering its effective mass or stiffness. In resonant sensing, the quantity to be measured is typically converted into one of these two parameters for measurement. Generally, in electric field measurements, the electrostatic force generated by induced charges causes a stiffness variation of ±Δk in the initial mechanical stiffness $k_{eff}$ of the resonator, as shown in Figure 14c. This variation enhances or reduces the overall stiffness, thereby altering the resonator’s initial Eigen-frequency. Considering the vibration of the resonator without stiffness perturbation, assume that the resonator is driven by a driving force F(t)=Fsin(ωt), where ω is the frequency of the driving force *F*. The displacement x(t) will also follow a harmonic curve. The equation of motion can be expressed as follows:

(19) $$m_{eff}\ddot{x}\left(t\right)+2\zeta \omega_{0}\dot{x}\left(t\right)+\omega_{0}^{2}x\left(t\right)=Fsin\left(\omega t\right)$$

Here, ζ represents the damping ratio of the vibration system, given by $\zeta =\frac{c_{eff}}{2m\omega_{0}}$, which can also be expressed as $\zeta =\frac{1}{2Q}$. The term Q refers to the Q-factor of the system, which is defined as:

(20) $$Q=2\pi \frac{Energy(average\;stored)}{Energy(lost\;per\;cycle)}=\frac{f_{0}}{\Delta f_{-3dB}}=\frac{m\omega_{0}}{c_{eff}}$$

where $\Delta f_{-3dB}$ denotes the −3 dB bandwidth centered around the resonant frequency. By solving Equation (19), the corresponding amplitude and phase responses of the resonator can be determined as:

(21) $$\left\{\begin{array}{c} x_{A}\left(\omega \right)=\frac{F/k}{\sqrt{{\left(1-{\left(\omega /\omega_{0}\right)}^{2}\right)}^{2}+4\zeta^{2}{\left(\omega /\omega_{0}\right)}^{2}}}\\ \phi \left(\omega \right)=arctan\left(\frac{2\zeta \omega /\omega_{0}}{1-{\left(\omega /\omega_{0}\right)}^{2}}\right) \end{array}\right.$$

**Figure 15:**
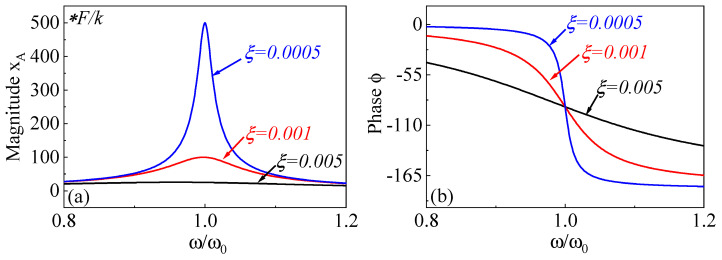
Amplitude (**a**) and phase (**b**) responses of a 1-DOF resonator under different damping ratios. A lower damping ratio corresponds to a higher Q-factor.

According to the analytical solutions for amplitude and phase, the amplitude–frequency and phase–frequency responses under normalized forcing frequency $\omega /\omega_{0}$ for different damping ratios are shown in Figure 15. When the driving frequency is near the Eigen-frequency, the amplitude–frequency response of the resonator is significantly larger than the static displacement $x_{A}\left(0\right)=F/k$. As shown in Figure 15, due to the presence of damping, the peak amplitude of the resonance does not occur exactly at the natural frequency but rather near it.

#### 3.1.2. Working Mechanism of the Sensor

The fundamental principle of EFSs based on a frequency shift output metric is that, under an applied electric field, induced charges are generated on axially or laterally perturbed electrodes, which cause a perturbation in the stiffness of the resonator. This perturbation leads to a change in the Eigen-frequency of the resonator. The frequency shift exhibits a linear relationship with the electric field input, thereby enabling the measurement of the electric field. As shown in Figure 14c, when a 1-DOF resonant system experiences a stiffness perturbation, the dynamic equation, considering the forced vibration with damping, can be expressed as:

(22) $$m_{eff}\ddot{x}\left(t\right)+c_{eff}\dot{x}\left(t\right)+\left(k+\Delta k\right)x\left(t\right)=F\left(t\right)$$

where Δk is the stiffness perturbation. In the Laplace domain, this equation is expressed as:

(23) $$m_{eff}s^{2}X\left(s\right)+c_{eff}sX\left(s\right)+\left(k_{eff}+\Delta k\right)X\left(s\right)=F\left(s\right)$$

This equation and corresponding transfer function can be derived as follows:

(24) $$\left\{\begin{array}{c} H(s)X(s)=F(s)\\ H\left(s\right)=m_{eff}s^{2}+c_{eff}s+\left(k_{eff}+\Delta k\right) \end{array}\right.$$

**Figure 16:**
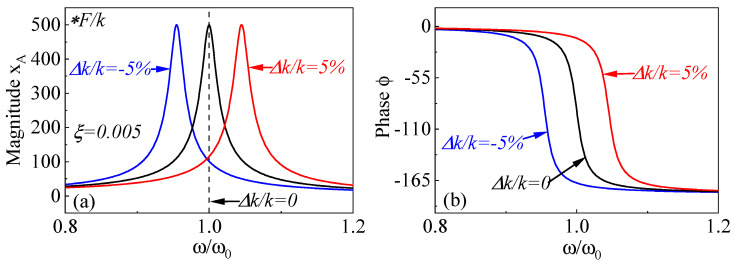
Amplitude (**a**) and phase (**b**) responses of a 1-DOF resonator under different stiffness adjustments.

The frequency response of the resonator under stiffness perturbation is solved using the transfer function, as shown in Figure 16. Under ±5% stiffness modulation, opposite spectrum shifts, including amplitude and phase responses, are observed. This indicates a significant opposite change in the resonant frequency depending on the positive or negative stiffness caused by the measured charge.

Assuming the driving force F(t) balances the system’s damping to maintain resonance, and considering a stiffness perturbation Δk, the frequency derived from solving Equation (22) is:

(25) $$\omega =\sqrt{\frac{k_{eff}+\Delta k}{m_{eff}}}$$

Since the stiffness perturbation Δk is much smaller than the effective stiffness *k* of the resonator, it follows that:

(26) $$\Delta \omega =\omega -\omega_{0}=\sqrt{\frac{k_{eff}+\Delta k}{m}}-\sqrt{\frac{k_{eff}}{m}}\approx \frac{\omega_{0}}{2}\frac{\Delta k}{k_{eff}}$$

The sensitivity of a frequency shift output sensor to stiffness perturbation is given by:

(27) $$S_{k}=\frac{\Delta \omega }{\Delta k}=\frac{\omega_{0}}{2k_{eff}}$$

### 3.2. Axial Stiffness Perturbation Method for Electric Field Measurement

**Figure 17:**
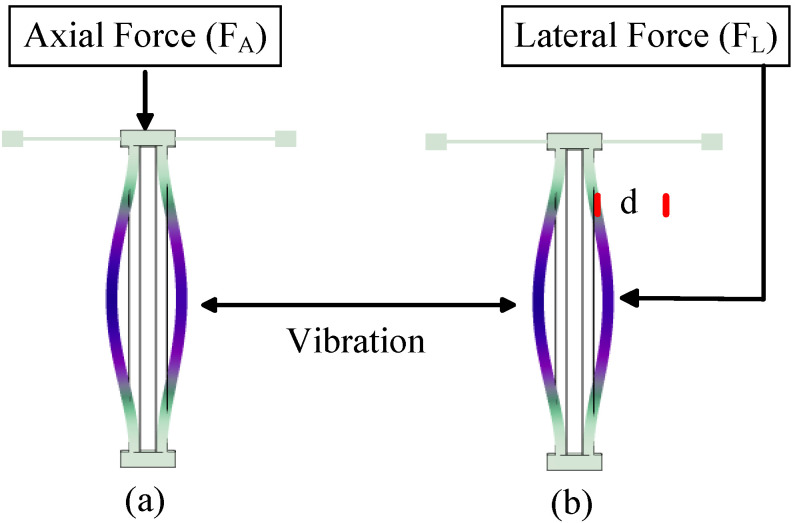
A simple design of a double-ended tuning fork resonant electrometer based on axial (**a**) and lateral (**b**) strain modulation schemes.

The axial force stiffness perturbation scheme is typically implemented using a bending resonant beam with a high length-to-width ratio to produce a significant axial direction under a uniformly applied force. Figure 17a shows a classic resonator structure for tuning stiffness with an axial force $F_{A}$, where one end of the double-beam resonator is fixed, and an axial force $F_{A}$ is applied to the other end, resulting in a change in the beam’s stiffness. By deriving the axial force generated in the bending vibration of the resonator, the change in stiffness of the resonator under axial force can be calculated:

(28) $$\Delta k_{axial}=\frac{12F_{A}}{\pi^{2}l}$$

where *l* is the length of the resonator, and its stiffness is:

(29) $$k_{mech}=\frac{192EI}{l^{3}}$$

where *E* is the Young’s modulus, and *I* is the moment of inertia. Therefore, the effective stiffness of the resonator is:

(30) $$k_{eff}=k_{mech}+\Delta k_{axial}=\frac{192EI}{l^{3}}+\frac{12EI}{\pi^{2}l}$$

Therefore, the influence of the axial force on the stiffness of the resonator increases its effective stiffness, raising the resonant frequency. This is a method of positive stiffness tuning.

**Figure 18:**
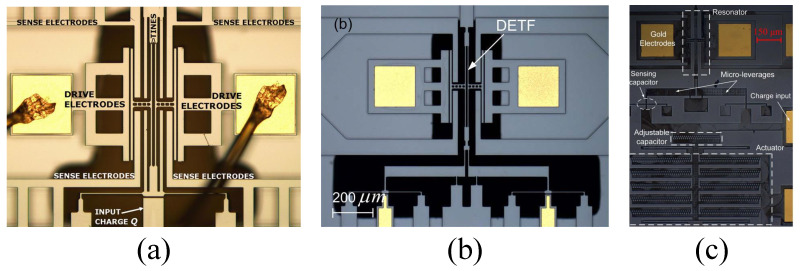
Microscope image of electrometer structure based on axial stiffness perturbation, with (**a**–**c**) corresponding to [47,48,73], respectively.

In 2008, J.E.-Y. Lee et al. reported a micromechanical electrometer [47] based on axial stiffness perturbation using a dual-end tuning fork resonator as a charge-sensing element, as shown in Figure 18a. The addition of charge to the input capacitor induces axial tensile strain in the resonator’s prongs, resulting in a change in the resonant frequency. Under a 3.0 V DC bias voltage and 4 mTorr pressure, the sensor exhibited a high Q-factor of 80,000 at a frequency of 154 kHz. When embedded in a closed-loop circuit, the resonator achieved a nonlinear oscillator with a short-term frequency deviation of 3.1 mHz (20 ppb). The noise-limited minimum detectable charge was 4 fC, corresponding to a noise force of 0.725 nN and a strain resolution of 23.4 pE.

As shown in Figure 18b, in 2015, J.X. Zhao et al. reported an electrostatic charge sensor based on MEMS resonator axial strain modulation [48]. This sensor uses a dual micro-lever design to enhance sensitivity. The amplification factor can be further increased by introducing multi-stage micro-lever structures. The core element of the sensor is a dual-end tuning fork resonator operating in an out-of-phase mode at 138.9 kHz, with a Q-factor of about 4900 under an operating pressure of 40 mTorr. The change in resonant frequency is proportional to the axial force induced by the added charge, which is then converted through a dual micro-lever with an amplification factor greater than eight. The measured response is $1.3\times 10^{-3}\;{\mathrm{Hz}/\mathrm{fC}}^{2}$, and the sensitivity is 21 fC with a frequency fluctuation of 4 ppm.

The schematic of the electrometer reported by D.Y. Chen et al. [73] in 2017 is shown in Figure 18c. This sensitivity modulation electrostatic measurement scheme is based on mechanical resonators and actuators. The device consists of a dual-end tuning fork resonator with symmetrically distributed levers and sensing capacitors and an adjustable capacitor controlled by a comb-drive actuator. The dual-end tuning fork resonator has a Q-factor close to 10,000 and a motion resistance below 0.5 MΩ. The charge-sensing function based on axial strain modulation provides a high resolution of 2.6 fC with a frequency fluctuation of 0.46 ppm. By adjustment, the sensitivity was linearized and further reduced, extending the dynamic range by 358.47% to 12.38 pC. Meanwhile, the frequency fluctuation remains stable below 70 mHz, demonstrating outstanding charge-sensing performance.

### 3.3. Lateral Stiffness Perturbation Method for Electric Field Measurement

Different from the changes in the mechanical properties of the beam under axial force, a force applied laterally from the direction of vibration can introduce a negative stiffness perturbation to the resonant system, thereby modulating the resonant frequency. For electrometry, the lateral stiffness perturbation scheme exhibits higher sensitivity and better performance, which will be illustrated in this section using beam resonators as examples. As shown in Figure 17b, in the lateral force stiffness perturbation scheme, the distance between the resonator and the electrode is *d*. Due to the gap and potential difference between the resonator and the fixed electrode, the electrode exerts an electrostatic attractive force on the resonator, and the magnitude of this electrostatic force is given by:

(31) $$F_{L}=\frac{1}{2}\Delta V^{2}\frac{\partial C_{d}}{\partial x}$$

where ΔV represents the potential difference between the two plates, $C_{d}=\epsilon_{0}A/d$ is the capacitance between the resonator and the electrode, *A* is the effective area between the resonator and the electrode, and *d* is the effective gap between the sensing electrode and the resonator. From the above expression, it can be seen that the electrostatic force is a displacement-dependent quantity. Therefore, according to Hooke’s law, the stiffness perturbation of the resonator due to the axial force input can be expressed as:

(32) $$\Delta k_{lateral}=-\frac{\partial F}{\partial x}=-\frac{\epsilon A\Delta V^{2}}{d^{3}}$$

Therefore, the effective stiffness of the resonator is the sum of the electrostatic negative stiffness and the mechanical stiffness of the resonator, i.e.:

(33) $$k_{eff}=k_{mech}+k_{lateral}=k_{mech}-\frac{\epsilon A\Delta V^{2}}{d^{3}}$$

The electrostatic negative stiffness effect is a method to soften the effective stiffness of a resonator. Electrostatic negative stiffness reduces the resonant frequency of the resonator, making it a negative tuning method for stiffness parameters. We can adjust the effective stiffness of the resonator by varying the potential difference ΔV between the resonator and the fixed electrode or by changing the gap *d* between the resonator and the electrode.

**Figure 19:**
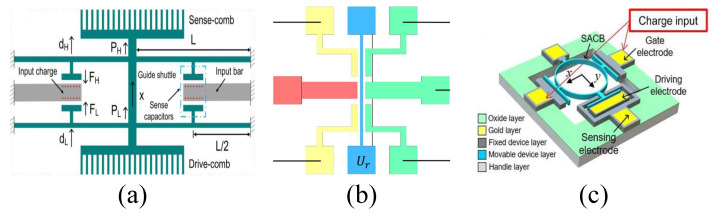
Lateral stiffness perturbation electrostatic measurement scheme based on a simple double-beam resonator (**a**), a single-beam resonator (**b**), and a single-ended anchored circular beam resonator (**c**) (modified from [49,74,75]).

In 2017, D.Y. Chen et al. reported a high-sensitivity resonant electrostatic charge-sensing scheme based on a simple twin-beam lateral stiffness perturbation [49], as shown in Figure 19a. The input charge generates a lateral electrostatic force that alters the effective stiffness of a double-ended tuning fork resonator, offering higher sensitivity than traditional axial strain sensing methods. The frequency sensitivity of the sensor is $4.4\times 10^{-4}$ Hz/fC^2^ and is almost unaffected by polarization voltage, with a relative frequency sensitivity of 9.1 ppb/fC^2^. By measuring the standard deviation of the output frequency, a charge resolution of 32.6 fC is obtained. This sensing scheme also creates an additional energy transfer path within the device, enhancing the Q-factor and stabilizing frequency fluctuations. Compared to the amplitude modulation method, the frequency modulation method shows better performance in terms of resolution and stability.

X.M. Liu et al. proposed a MEMS EFS based on a simple single-beam structure with lateral strain modulation [75] in 2023, as shown in Figure 19b. This sensor utilizes the resonant frequency as the output signal to eliminate feedthrough interference from the driving voltage. The sensor consists of a resonator, driving electrode, sensing electrode, transition electrode, and electric field sensing plate. Its working principle is that when an electric field is present, induced charges appear on the surface of the sensing plate, generating electrostatic stiffness in the resonator, which causes a shift in the resonant frequency. Experimental results show that the sensor has a sensitivity of 0.1384 $\sqrt{\mathrm{Hz}}$/(kV/m) and a resolution better than 10 V/m.

The schematic of the MEMS frequency-modulated electrometer prototype based on a pre-bias charging mechanism [74] reported by H.Y. Chen et al. in 2022 is shown in Figure 19c. This electrometer is a lateral-force stiffness perturbation scheme based on a single-anchor circular beam resonator structure for electrostatic charge measurement. The single-anchor circular beam design overcomes the issue of uneven energy distribution in the traditional axially extended tuning fork structures and reduces geometric nonlinearity. Experimental results show that as the pre-bias charge increases, the resonator transitions from a low-sensitivity to a high-sensitivity regime. In open-loop measurements, the sensitivity of the SACB electrometer is 5.14 ppm/fC at a bias of 1.416 pC, while in closed-loop measurements, the sensitivity is 4.52 ppm/fC. As the bias increases from 0.708 pC to 1.416 pC, the charge resolution improves nearly 20 times, and the dynamic range expands by 131%. The pre-bias mechanism can also be applied to other resonant sensing applications to enhance performance.

## 4. Mode-Localized Electric Field Microsensors

Mode localization refers to a phenomenon in which, in a mechanically identical and symmetric coupled resonator system, the vibrational energy of a given mode is evenly distributed across the resonators. The vibration mode extends uniformly across all resonators when the system maintains dynamic symmetry. However, when the coupling coefficient between the resonators is small, and a perturbation occurs in the mechanical parameters of one resonator, breaking the system’s symmetry, the vibrational energy becomes confined to a single resonator. This energy confinement can be measured through eigenstate or amplitude ratio analysis, known as mode localization. This section introduces the theoretical analysis and principles of mode localization and reviews prototype instantiations of its application in charge and electric field measurements.

### 4.1. Theoretical Analysis

Several different methods have been previously studied and implemented to model weakly coupled mode-localized systems and strongly coupled multi-DoF systems. Although these methods remain effective in modeling the mechanical behavior of such systems, their utility is limited in constructing integrated models for practical applications because MEMS devices require models that can capture both mechanical and electrical behaviors, including electronic interfaces. This section evaluates three modeling approaches applicable to mode-localized resonator systems: (1) eigenvalue analysis, which predicts the eigenvalues and eigenvectors of a coupled system under perturbation; (2) the transfer function method, which simplifies the mechanical dynamics of resonators into a transfer function to predict the amplitudes of two resonators under different perturbation states; and (3) the BVD electrical model, which enables the resonators to be integrated into systems with readout electronics and closed-loop oscillator circuits. The combination of these three methods helps to predict the behavior of practical MEMS sensors more accurately.

#### 4.1.1. Eigenvalue Analysis

**Figure 20:**
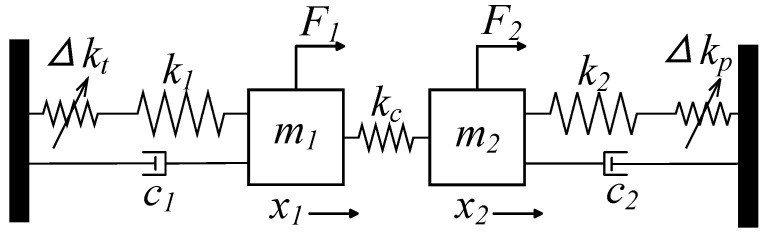
Lumped spring-mass-dashpot model of 2-DOF coupled system.

Mode localization can be observed in a lumped model consisting of multiple 1-DoF micromechanical resonators connected by weak coupling springs. Considering a 2-DOF weakly coupled resonator as an example, its spring-mass-dashpot model is shown in Figure 20. In this model, the resonators have masses $m_{1}$ and $m_{2}$, stiffnesses $k_{1}$ and $k_{2}$, and damping coefficients $c_{1}$ and $c_{2}$. It is assumed that these parameters are similar (i.e., $m_{1}$ = $m_{2}$ = *m*, $k_{1}$ = $k_{2}$ = *k*, $c_{1}$ = $c_{2}$ = *c*), and the stiffness of the coupling spring is much weaker than that of the resonators themselves (i.e., $k_{c}\ll k$). Disorder in the system is introduced by adjusting the stiffness of one of the resonators, represented as a perturbation $\Delta k_{p}$ on one of the springs, while $\Delta k_{t}$ is used to adjust for system imbalance caused by process errors. It should be noted that in practical sensors, $\Delta k_{p}$ serves as a proxy for the measurement. In the absence of damping and external forces, the equations of motion for the system can be described as follows:

(34) $$\left\{\begin{array}{c} m\ddot{x_{1}}+\left(k+k_{c}\right)x_{1}-k_{c}x_{2}=0\\ m\ddot{x_{2}}+\left(k+k_{c}+\Delta k_{p}\right)x_{2}-k_{c}x_{1}=0 \end{array}\right.$$

It can be expressed in matrix form as:

(35) $$\left[\begin{array}{cc} m & 0\\ 0 & m \end{array}\right]\left[\begin{array}{c} {\ddot{x}}_{1}\\ {\ddot{x}}_{2} \end{array}\right]+\left[\begin{array}{cc} k+k_{c} & -k_{c}\\ -k_{c} & k+\Delta k_{p}+k_{c} \end{array}\right]\left[\begin{array}{c} {\ddot{x}}_{1}\\ {\ddot{x}}_{2} \end{array}\right]=\left[\begin{array}{c} 0\\ 0 \end{array}\right]$$

Assuming harmonic displacement, ${\left[x_{1}x_{2}\right]}^{T}=u_{n}e^{\left(i\omega t\right)}\left(n=1,2\right)$, the eigenvalues of the system can be found by setting the determinant of the system as equal to zero.

(36) $$\left|\begin{array}{cc} -\omega^{2}m+k+k_{c} & -k_{c}\\ -k_{c} & -\omega^{2}m+k+\Delta k_{p}+k_{c} \end{array}\right|=0$$

The eigenvalues and eigenvectors of the system can be obtained.

(37) $$\left\{\begin{array}{c} \omega_{i}^{2}=\frac{2k+2k_{c}+\Delta k_{p}\pm \sqrt{\Delta k_{p}^{2}+4k_{c}^{2}}}{2m}\left(i=1,2\right)\\ u_{1}=\frac{x_{i2}}{x_{i1}}=\frac{\Delta k_{p}\mp \sqrt{\Delta k_{p}^{2}+4k_{c}^{2}}}{2k_{c}}\left(i=1,2\right) \end{array}\right.$$

Assuming $\Delta k_{p}=0$ in the unperturbed case, the eigenvalues, eigenvectors, and amplitude ratio of the two modes are as follows:

(38) $$\left\{\begin{array}{c} \omega_{01}^{2}=\frac{k}{m};\;\omega_{02}^{2}=\frac{k+2k_{c}}{m};\\ u_{01}=\frac{1}{\sqrt{2}}\left[1;1\right];\;u_{02}=\frac{1}{\sqrt{2}}\left[1;-1\right];\\ AR_{01}=1;\;AR_{02}=-1; \end{array}\right.$$

Define the relative sensitivity as the rate of relative frequency change and AR per unit input. For mode 1, the relative frequency change rate and the relative AR change rate can be expressed as:

(39) $$V_{\omega 1}=\frac{\omega_{1}-\omega_{0}}{\omega_{0}}\approx \frac{1}{4}\left(2\kappa +\Delta k_{p}-\sqrt{\Delta k_{p}^{2}+4\kappa^{2}}\right)$$

(40) $$V_{u1}=\frac{u_{1}-1}{1}=\frac{1}{2\kappa }\left(-\Delta k_{p}+\sqrt{\Delta k_{p}^{2}+4\kappa^{2}}-2\kappa \right)$$

where $\kappa =k_{c}/k$ is the coupling coefficient. Then, we obtain the sensitivity of the frequency and amplitude ratio to stiffness changes as:

(41) $$\left\{\begin{array}{c} S_{\omega 1}=\frac{\partial V_{\omega 1}}{\partial \Delta k_{p}}=\frac{1}{4}\left(1-\frac{\Delta k_{p}}{\sqrt{\Delta k_{p}^{2}+4\kappa^{2}}}\right)\\ S_{AR1}=\frac{\partial V_{u1}}{\partial \Delta k_{p}}=\frac{1}{2\kappa }\left(-1+\frac{\Delta k_{p}}{\sqrt{\Delta k_{p}^{2}+4\kappa^{2}}}\right) \end{array}\right.$$

Then, the relative value of AR sensitivity to frequency sensitivity is:

(42) $$\left|\begin{array}{c} \frac{S_{AR1}}{S_{\omega 1}} \end{array}\right|\approx \frac{2}{\kappa }=\frac{2k}{k_{c}}$$

For weak coupling, where $k_{c}\ll k$, we can conclude that the AR sensitivity is much higher than the frequency sensitivity. This is the fundamental principle behind the significant sensitivity improvement in weakly coupled resonant sensors based on mode localization.

#### 4.1.2. Transfer Function Analysis

In a weakly coupled mode-localized resonant system, we can construct transfer function equations to integrate mechanical and electrical behavior, enabling an in-depth analysis of the system’s overall dynamic characteristics. This method can simulate the variation in vibration amplitude under input disturbances and accurately describe the complex noise processes within the system. The model of the 2-DOF weakly coupled system can be established using the forced vibration dynamic model shown in Figure 20. Assuming a single-end driving approach ($F_{2}=0$, the equation for the forced vibration of the resonant system is:

(43a) $$\begin{array}{c} m{\ddot{x}}_{1}+c{\dot{x}}_{1}+\left(k+k_{c}\right)x_{1}-k_{c}x_{2}=F_{1}\left(t\right) \end{array}$$

(43b) $$\begin{array}{c} m{\ddot{x}}_{2}+c{\dot{x}}_{2}+\left(k+k_{c}+\Delta k_{p}\right)x_{2}-k_{c}x_{1}=0 \end{array}$$

The equation can be expressed in the Laplace domain as:

(44a) $$\begin{array}{c} ms^{2}x_{1}\left(s\right)+csx_{1}\left(s\right)+\left(k_{1}+k_{c}\right)x_{1}\left(s\right)=F_{1}\left(s\right)+k_{c}x_{2}\left(s\right) \end{array}$$

(44b) $$\begin{array}{c} ms^{2}x_{2}\left(s\right)+csx_{2}\left(s\right)+\left(k_{2}+k_{c}+\Delta k_{p}\right)x_{2}\left(s\right)=k_{c}x_{1}\left(s\right) \end{array}$$

This can be simplified to:

(45a) $$\begin{array}{c} H_{1}\left(s\right)x_{1}\left(s\right)=F_{1}\left(s\right)+k_{c}x_{2}\left(s\right) \end{array}$$

(45b) $$\begin{array}{c} H_{2}\left(s\right)x_{2}\left(s\right)=k_{c}x_{1}\left(s\right) \end{array}$$

where $H_{1}\left(s\right)=ms^{2}+cs+\left(k_{1}+k_{c}\right)$ and $H_{2}\left(s\right)=ms^{2}+cs+\left(k_{1}+k_{c}+\Delta k_{p}\right)$ are the transfer functions. The displacement response of each resonator to each applied force can be derived using Cramer’s rule as follows:

(46) $$\left[\begin{array}{cc} H_{1}\left(s\right) & -k_{c}\\ -k_{c} & H_{2}\left(s\right) \end{array}\right]\left[\begin{array}{c} X_{1}\left(s\right)\\ X_{2}\left(s\right) \end{array}\right]=\left[\begin{array}{c} F_{1}\left(s\right)\\ 0 \end{array}\right]$$

(47) $$x_{1}=\frac{\left|\begin{array}{cc} F_{1}\left(s\right) & -k_{c}\\ 0 & H_{2}\left(s\right) \end{array}\right|}{\left|\begin{array}{cc} H_{1}\left(s\right) & -k_{c}\\ -k_{c} & H_{2}\left(s\right) \end{array}\right|};x_{2}=\frac{\left|\begin{array}{cc} H_{1}\left(s\right) & F_{1}\left(s\right)\\ -k_{c} & 0 \end{array}\right|}{\left|\begin{array}{cc} H_{1}\left(s\right) & -k_{c}\\ -k_{c} & H_{2}\left(s\right) \end{array}\right|};$$

(48) $$x_{1}=\frac{F_{1}\left(s\right)H_{2}\left(s\right)}{H_{1}\left(s\right)H_{2}\left(s\right)-k_{c}^{2}};x_{2}=\frac{F_{1}\left(s\right)k_{c}}{H_{1}\left(s\right)H_{2}\left(s\right)-k_{c}^{2}};$$

**Figure 21:**
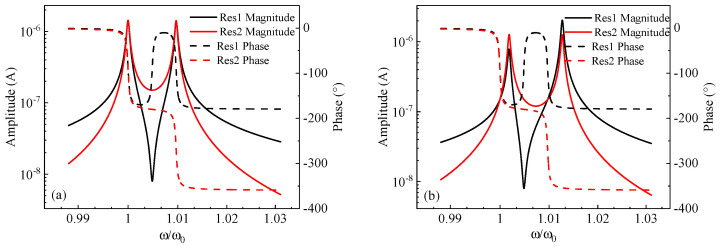
Frequency response of a 2-DOF weakly coupled resonant system in both symmetrical (**a**) and asymmetrical (**b**) conditions, obtained using the transfer function equation.

Based on the transfer function of a weakly coupled resonant system, the frequency response of two resonators under a single-end drive with stiffness perturbation can be simulated. As shown in Figure 21, without stiffness perturbation, the two resonators are in symmetric resonance. After applying stiffness perturbation, the resonators shift to an asymmetric state, clearly showing the mode localization phenomenon. Additionally, the transfer function equation can also be used to simulate the frequency response under a double-end drive.

#### 4.1.3. Butterworth–Van Dyke Model Analysis

**Figure 22:**
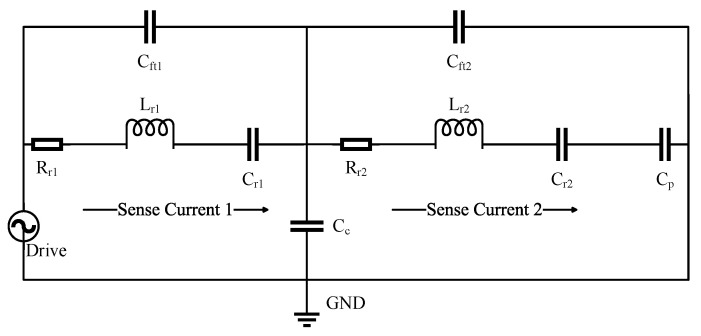
BVD model corresponding to a 2-DOF weakly coupled resonant system.

By coupling the two 1-DOF resonators shown in Figure 5 through a coupling capacitor component, the mechanical model in Figure 20 can be transformed into a BVD model to analyze the effective output current of the 2-DOF weakly coupled micromechanical resonators. The coupling capacitance must be very small to ensure the weak coupling of the two resonator systems. The equivalent RLC circuit, including two parasitic capacitors, is shown in Figure 22. Based on the deductions in Section 2.2 regarding the equivalent RLC circuit of a 1-DOF resonator, the output currents of the two resonators in the 2-DOF weakly coupled mode-localized resonator system at their eigenfrequency points are:

(49a) $$\begin{array}{c} i_{s1}\propto \frac{1}{\Xi }\left[\left(2+\frac{k^{2}C_{ft1}^{2}}{Q\eta^{2}}\right)+\frac{jk(\Theta -\frac{Q\eta^{2}C_{ft2}}{m})}{\sqrt{Q}\eta }\right] \end{array}$$

(49b) $$\begin{array}{c} i_{s2}\propto \frac{1}{\Xi }\left[\left(2+\frac{k^{2}C_{ft1}^{2}}{Q\eta^{2}}\right)+\frac{jk\Theta }{\sqrt{Q}\eta }\right] \end{array}$$

where $\Theta =4C_{ft2}+C_{ft1}-\frac{k^{2}C_{ft1}^{2}C_{ft2}}{Q\eta^{2}}$, and $\Xi =R_{r}\left(1+\frac{L_{r}R_{r}^{2}C_{ft1}^{2}}{C_{r}}\right)$. The analysis of the output currents of the two resonators reveals the following effects of feedthrough capacitance on the weakly coupled resonant system: (1) when driving resonator 1, the real part of the output current from both resonators is only affected by the feedthrough capacitance of resonator 1, while the feedthrough capacitance of both resonators influences the imaginary part; (2) the presence of feedthrough capacitance causes the resonant frequency points of the two resonators that are not to coincide, which introduces errors when measuring the AR, severely affecting the accuracy of weakly coupled resonant sensors. These effects are critical for mode-localized resonant sensors, and it is essential to account for the impact of feedthrough capacitance in chip design, taking measures to eliminate or minimize its influence.

### 4.2. Prototype Instantiation for Mode-Localized Microsensor

Various structures, methods, and features of mode-localization have been employed to enhance the performance of weakly coupled sensors, including multi-DOFs and nonlinearity [76,77]. Different structures and multi-DOFs result in varying performance for mode-localized EFSs and electrometers. To illustrate the current performance of mode-localized EFSs and electrometers and to clarify the future applications of this technology, this paper discusses the most common parameters of mode-localized electrometers and EFSs, such as sensitivity, bias instability, noise spectral density, resolution, measurement range, and bandwidth.

#### 4.2.1. A 2-DOF Mode-Localized Resonant Microsensor

**Figure 23:**
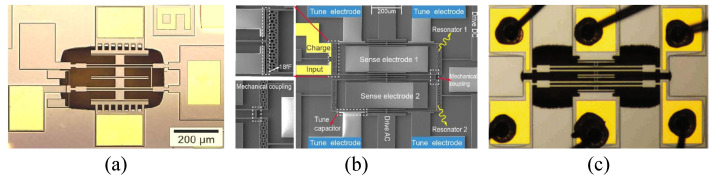
Implementation of 2-DOF weakly coupled mode-localized resonant electrometers (**a**,**b**) corresponding to [18,51] and charge sensors (**c**) [78].

In 2010, P. Thiruvenkatanathan et al. first reported an ultrasensitive prototype mode-localized micromechanical electrometer [18], utilizing the phenomena of mode-localization and curve veering to detect small changes in charge on the input electrode. As shown in Figure 23a, this electrometer consists of two nearly identical double-ended tuning fork (DETF) resonators connected by a weakly coupled mechanical beam. The input charge induces a differential axial stiffness perturbation between the two resonators, leading to mode-localization. Experimental results demonstrate that for the same charge input, the resulting eigenstate shifts are nearly three orders of magnitude larger than the corresponding changes in resonant frequency, which is consistent with the theoretical predictions.

In 2016, H.M. Zhang et al. reported a high-sensitivity resonant electrometer based on the mode-localization phenomenon [51], as shown in Figure 23b. This electrometer measures changes in the input charge by detecting variations in the AR. Experimental results indicate that the relative AR sensitivity is 663,751 ppm, which is 2341 times greater than the relative frequency shift sensitivity of 283.56 ppm. In the same year, H.M. Zhang et al. conducted a more comprehensive performance characterization of the structure. They developed a theoretical model of the electrometer through transfer functions and established design rules for the coupling factor based on the −3 dB bandwidth, AR measurement error, and frequency mismatch between the resonators. Experimental results showed that the AR sensitivity was 2151 times higher than the frequency shift sensitivity, achieving an AR-based resolution of 1.269 fC. The resolution can be further improved by enhancing sensitivity and reducing amplitude noise, while optimizing device topology and operating pressure to increase the Q-factor is highly beneficial for noise reduction.

The schematic of the novel ultrasensitive single-electron detection charge sensor [78] reported by X.F. Wang et al. is shown in Figure 23c. This charge sensor utilizes nonlinearity to significantly enhance resolution and sensitivity, revealing the great potential of nonlinear applications. A real-time closed-loop measurement circuit was developed for charge detection. Experimental results demonstrated that the sensor achieved single-electron charge detection at room temperature, with a resolution of 0.197 ± 0.0056 e/$\sqrt{\mathrm{Hz}}$.

#### 4.2.2. Multi-DOF Mode-Localized Microsensor

Unlike conventional 2-DOF mode-localized EFSs and electrometers, multi-DOF mode-localized resonant EFSs and electrometers are achieved by increasing the number of weakly coupled resonators. Adding more resonators to the weakly coupled system effectively improves the sensitivity and resolution and enables additional functionalities [50,79,80,81,82,83]. This section describes the 3-DOF and 4-DOF structures of mode-localized electrometers and EFSs.

**Figure 24:**
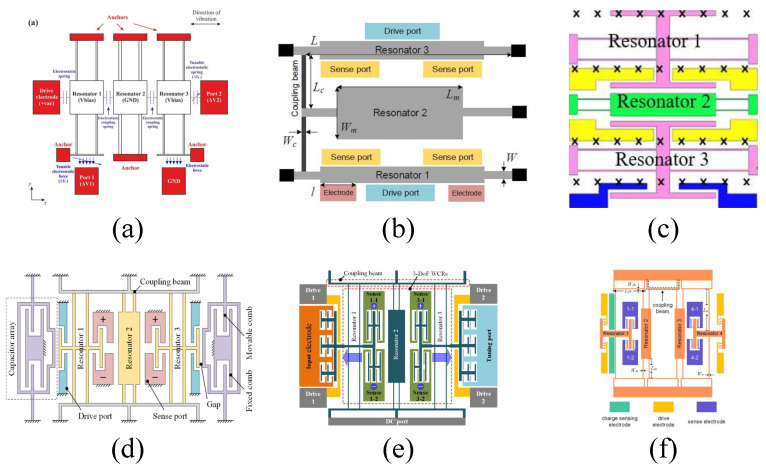
Schematic diagrams of 3-DOF and 4-DOF (**f**) weakly coupled mode-localized resonant EFSs and electrometers, with (**a**–**f**) corresponding to [50,79,80,81,82,83], respectively.

In 2017, C. Zhao et al. reported the first novel 3-DOF mode-localized MEMS electrical potential sensor [50]. As shown in Figure 24a, the sensor consists of three mechanical resonators placed side-by-side and coupled through capacitive coupling. Unlike the 2-DOF structure, resonators 1 and 3 are used for potential detection, while the middle resonator 2 is weakly coupled to the two outer resonators via capacitors. Based on the 3-DOF mode localization, the AR sensitivity is:

(50) $$S_{3DOF}=\left|\begin{array}{c} \frac{\partial (AR)}{\partial (\Delta k/k)} \end{array}\right|=\frac{k(k_{2}-k+k_{c})}{k_{c}^{2}}$$

where *k*, $k_{2}$, and $k_{c}$ represent the stiffness of the suspension beam of resonator 1 (and 3), resonator 2, and the coupling spring, respectively, with $k_{2}>2k$. Compared to the 2-DOF mode-localized AR sensitivity in equation 42, the sensitivity of this design can be increased by a factor of $\frac{k_{2}-k+k_{c}}{4k_{c}}$. The 3-DOF design demonstrates an improvement of two orders of magnitude in sensitivity. Moreover, experimental results show that the maximum sensitivity is over 123 times higher than the most advanced 2-DOF mode-localized sensors. The noise floor for potential sensing is estimated to be 614 $\mu V/\sqrt{\mathrm{Hz}}$, while the noise floor for charge sensing is 57.6 $e/\sqrt{\mathrm{Hz}}$, with a dynamic range of up to 66.3 dB.

J. Yang et al. reported a micro resonant electrometer in 2018, achieving a resolution of nine electrons at room temperature. As shown in Figure 24b, the electrometer utilizes a 3-DOF weakly coupled resonator as the sensing element, with the input charge-inducing mode localization. Closed-loop test results showed that the relative AR sensitivity was 900 times higher than the relative frequency shift sensitivity, with a resolution of 9.21 e$/\sqrt{\mathrm{Hz}}$.

Z.L. Wang et al. proposed a novel high-sensitivity mode-localized EFS structure [84] based on closed-loop feedback, as shown in Figure 24c. Test results under vacuum conditions demonstrated that this EFS achieved a DC electric field resolution of 10 V/m, 5.3% accuracy, and 0.56% repeatability (2021). Subsequently, X.M. Liu et al. reported a sensitivity and stability-enhanced EFS based on this structure. The mode-localized EFS operates at 15,893 Hz and 15,896 Hz in in-phase and out-of-phase eigenmodes, achieving an electric field sensitivity of 0.0032 /(V/m) and a resolution better than 10 V/m.

The schematic of the micro-resonant DC EFSs [80] based on the mode localization phenomenon reported by Z.M. Yan in 2019 is shown in Figure 24d. The sensor employs a 3-DOF weakly coupled resonator as the mechanical sensing element, with comb capacitors on both sides to convert the electric field into stiffness perturbations. Experimental results within the measurement range of 7 kV/m demonstrate that the AR-based sensitivity is 1720 times higher than that based on frequency shift, with a resolution of 20.4 V/m$\sqrt{\mathrm{Hz}}$. In 2022, Y.C. Hao et al. reported a mode-localized DC EFS based on this structure [83]. Within the electric field range of 0–7 kV/m, a sensitivity of 0.76 /(kV/m) was measured. The noise was 11.5 (V/m)/$\sqrt{\mathrm{Hz}}$, the resolution was 22.9 V/m, and the stability was 9.1 V/m.

In 2021, Y.C. Hao et al. reported a high-resolution voltmeter based on the application of mode localization in a 3-DOF weakly coupled resonator system [82], as shown in Figure 24e. The input voltage induces stiffness perturbations in the resonators, leading to mode localization and enabling voltage detection. The sensor operates at eigenmodes of 19,626 Hz and 19,639 Hz, with a measured Q-factor of 32,000. The AR sensitivity is 2918 times higher than the frequency sensitivity. The bias instability is 42.6 μV, AR noise is $1.02\times 10^{-4}/\sqrt{\mathrm{Hz}}$, resolution reaches 3 $\mu V/\sqrt{\mathrm{Hz}}$, and both repeatability and hysteresis errors are below 3%.

In 2020, H. Kang et al. reported the first novel 4-DOF micromachined electrometer with room temperature resolution of 0.256 e/$\sqrt{\mathrm{Hz}}$ [81]. As shown in Figure 24f, the sensor consists of four mechanical resonators placed side-by-side and coupled through capacitors. Unlike the 2-DOF structure, resonators 1 and 4 are used for potential detection, while resonators 2 and 3 are weakly coupled to the outer resonators through capacitors. The mode-localized AR sensitivity based on the 4-DOF configuration is:

(51) $$S_{4DOF}=\left|\begin{array}{c} \frac{\partial (\Delta u/u_{0})}{\partial (\Delta k/k)} \end{array}\right|\approx \frac{5k_{m}^{2}k-9k_{m}k^{2}+4k^{3}}{5k_{c}^{3}}$$

where $k_{m}$ is the stiffness of resonators 2 and 3. Compared to the 2-DOF mode-localized AR sensitivity in Equation (42), the sensitivity of this design can be increased by a factor of $\frac{5k_{m}^{2}-9k_{m}k+4k^{2}}{10k_{c}^{2}}$. The 4-DOF design demonstrates an improvement of orders of magnitude in sensitivity. The structure operates at eigenmodes of 14,569.95 Hz and 14,571.56 Hz under room temperature conditions, featuring a measurement range of over 500,000 electrons, a dynamic range of 136.7 dB, a sensitivity of 0.047 V/fC, and a resolution of 0.256 e/$\sqrt{\mathrm{Hz}}$.

## 5. Discussion

This paper conducts a comprehensive overview of various resonant electric field micro-sensors, including micro-field-mills, micro-cantilever EFSs, frequency-modulated EFSs, and mode-localized EFSs. These sensors have been successfully used in atmospheric electric field measurements and static charge detection.

In electric field sensors, different working principles and sensing structures significantly impact sensitivity, primarily depending on the structural design and the sensor’s ability to sense charges. For in-plane vibrating micro-field-mills based on charge induction, parallel plate capacitive sensing and comb-finger capacitive sensing are common configurations. The parallel plate capacitive structure is simple in design and offers good stability, but its sensitivity is limited due to the restricted electrode area. In contrast, the comb-finger structure increases the sensing area by adding multiple interdigitated electrodes (combs), thus significantly enhancing the sensitivity for electric field measurement. Table 2 also highlights the high sensitivity characteristic of comb-finger sensing in micro-field-mills.

Torsional vibrating electric field sensors, compared to in-plane vibrating ones, demonstrate higher charge-sensing efficiency, resulting in greater sensitivity, as reflected in the performance parameter comparison in Table 2. However, in terms of fabrication, in-plane vibrating electric field sensors can be directly realized through electrostatic actuation and detection, making them easier to produce compared to torsional vibrating sensors. In comparison to both of these types, rotational vibrating electric field sensors offer unique advantages, as they achieve dual-axis electric field detection while maintaining high sensitivity and a wide measurement range on a single chip. In the future, realizing single-chip three-dimensional electric field measurements could become a key research direction.

The frequency-modulated and mode-localized electric field sensors are based on stiffness perturbations in the resonators caused by external electric fields. Frequency-modulated EFS typically requires specially designed sensing structures to enhance charge detection capabilities or improve the quality factor to enhance sensor sensitivity. However, mode-localized EFS, which relies on amplitude ratio output, exhibits significant amplitude ratio changes when the coupling structure in the system is perturbed. Compared to frequency-modulated sensors, mode-localized sensors generally offer higher sensitivity by measuring the amplitude ratio of coupled resonators. Additionally, they demonstrate superior common-mode rejection and environmental noise immunity.

## 6. Conclusions

This review provides an analysis of the principles and performances of resonant MEMS EFSs over recent decades, with a particular focus on their structural characteristics and resonance behaviors. Table 2 compares the performance metrics of MEMS EFS with different working principles and sensing structures. Research on micro-field-mills has shown that they feature various sensitive structures and vibration modes, achieving high sensitivity and wide measurement ranges, and they have been successfully applied in power-grid monitoring and atmospheric electric field measurements. Although there has been less research on frequency-modulated electric field sensors, the optimization of sensitive structures has led to improvements in sensitivity and other performance metrics. Mode-localized electric field sensors, based on amplitude ratio output, significantly enhance sensitivity and resolution. These performance improvements are primarily attributed to their structural design and coupling mechanisms. While mode-localized electric field sensors have not yet seen broad application in electric field measurements, further research may enable their future use. Utilizing parametric actuation and nonlinearity can significantly improve the performance of mode-localized sensors, enhancing their sensitivity and energy exchange capabilities, which could foster further studies in this area. Currently, there are few reports on electric field sensors based on these principles, but their potential for highly sensitive electric field measurements in industrial applications is promising.

## Figures and Tables

**Table 1:** Parameters of the mechanical system and their corresponding electrical system parameters.

Mechanical System	Electrical System
Force (*F*)	Voltage (*V*)
Velocity (*v*)	Current (*I*)
Stiffness (*k*)	Capacitance (*C*)
Mass (*m*)	Inductance (*L*)
Damping (*c*)	Resistance (*R*)

**Table 2:** The performance of resonant MEMS EFS with different structures and principles.

	Sensitivity	Measurement Range	Structural Characteristics	Resolution
Mirco Field Mill				
EFS by Peng et al. [24]	-	0–50 kV/m	parallel plate sense	100 V/m
EFS by Wen et al. [70]	-	−1–+1 kV/m	comb-finger sense	5 V/m
EFS by Liu et al. [63]	0.16 mV/(kV/m)	0–50 kV/m	parallel plate sense	-
EFS by Bahreyni et al. [32]	0.16 mV/(kV/m)	0–5 kV/m	parallel plate sense	42 V/m
Charge sensor by Zhu et al. [33]	1.58 nV /e	-	parallel plate sense	68.3 e/$\sqrt{\mathrm{Hz}}$
EFS by Gao et al. [67]	0.31 mV/(kV/m)	0–50 kV/m	parallel plate sense	230 V/m
EFS by Wen et al. [46]	465 mV/(kV/m)	0–100 kV/m	comb-finger sense	10 V/m
EFS by Chu et al. [30]	4.82 mV/(kV/m)	0–50 kV/m	torsional resonant and parallel plate sense	-
EFS by Wang et al. [28,29]	0.675 mV/(kV/m)	0–25 kV/m	rotary resonant and comb-finger sense	-
EFS by Huang et al. [71]	0.84 mV/(kV/m)	0–1 kV/m	micro-cantilever	-
EFS by Han et al. [72]	-	1.1–1100 kV/m	micro-cantilever	112 V/m/$\sqrt{\mathrm{Hz}}$
Frequency-Modulated				
Electrometer by Lee et al. [47]	-	0–200 fC	axial strain modulated and tuning fork	4 fC
Electrometer by Chen et al. [73]	-	12.38 pC	adjustable capacitor and tuning fork	2.6 fC
EFS by Liu et al. [75]	0.1384 $\sqrt{\mathrm{Hz}}$/(kV/m)	0–10 kV/m	lateral modulated and double-clamped beam	10 V/m/$\sqrt{\mathrm{Hz}}$
Mode-localized				
Electrometer by Zhang et al. [51,52]	663,751 ppm/C	0–144 fC	weakly coupled tuning fork	1.269 fC
electrical potential sensor by Zhao et al. [50]	-	−6–+2 V	three electrically coupled resonators	614 $\mu V/\sqrt{\mathrm{Hz}}$
Voltmeter by Hao et al. [82]	34/V	0–0.25 V	three mechanically coupled resonators	3 $\mu V/\sqrt{\mathrm{Hz}}$
EFS by Liu et al. [85]	3.2/(kV/m)	0–11 kV/m	three electrically coupled resonators	10 V/m/$\sqrt{\mathrm{Hz}}$
EFS by Hao et al. [83]	0.76/(kV/m)	0–7 kV/m	three mechanically coupled resonators	11.5 V/m/$\sqrt{\mathrm{Hz}}$
